# Quantitative and simultaneous translational control of distinct mammalian mRNAs

**DOI:** 10.1093/nar/gkt347

**Published:** 2013-05-18

**Authors:** Kei Endo, James A. Stapleton, Karin Hayashi, Hirohide Saito, Tan Inoue

**Affiliations:** ^1^International Cooperative Research Project, Japan Science and Technology Agency, 5 Sanban-cho, Chiyoda-ku, Tokyo 102-0075, Japan, ^2^Department of Reprogramming Science, Center for iPS Cell Research and Application, Kyoto University, 53 Kawahara-cho, Shogoin, Sakyo-ku, Kyoto 606-8507, Japan, ^3^Laboratory of Gene Biodynamics, Graduate School of Biostudies, Kyoto University, Oiwake-cho, Kitashirakawa, Sakyo-ku, Kyoto 606-8502, Japan and ^4^The Hakubi Center for Advanced Research, Kyoto University, Oiwake-cho, Kitashirakawa, Sakyo-ku, Kyoto 606-8502, Japan

## Abstract

The introduction of multiple genes into cells is increasingly required for understanding and engineering biological systems. Small-molecule–responsive transcriptional regulation has been widely used to control transgene expression. In contrast, methods for specific and simultaneous regulation of multiple genes with a single regulatory protein remain undeveloped. In this report, we describe a method for quantitatively tuning the expression of multiple transgenes with a translational regulatory protein. A protein that binds a specific RNA motif inserted in the 5′-untranslated region (UTR) of an mRNA modulates the translation of that message in mammalian cells. We provide two independent mechanisms by which to rationally fine-tune the output: the efficiency of translation correlates well with the distance between the inserted motif and the 5′ terminus of the mRNA and is further modulated by the tandem insertion of multiple RNA motifs. The combination of these two approaches allowed us to fine-tune the translational efficiency of target mRNAs over a wide dynamic range. Moreover, we controlled the expression of two transgenes simultaneously and specifically by engineering each *cis*-regulatory 5′-UTR. The approach provides a useful alternative regulatory layer for controlling gene expression in biological research and engineering.

## INTRODUCTION

Construction of synthetic gene regulatory systems that quantitatively and simultaneously control expression of multiple genes is important for both understanding and engineering biological processes. Given that the complexity of the gene regulatory systems being constructed and integrated into cells is increasing rapidly (1–4), the expression of the exogenous genes that comprise these synthetic circuits must be controlled precisely to ensure optimal system performance. The independent regulation of multiple exogenous genes in a single cell is particularly gaining in importance. For example, ectopic expression of four transcription factors can induce pluripotency in adult somatic cells (5). Another specific set of factors can convert exocrine cells in the mouse pancreas into insulin-producing endocrine cells (6). The stoichiometry of these factors has an impact on the efficiency of reprogramming (7). However, artificially introduced transgenes are generally uncontrolled in comparison with their strictly programmed, genomically encoded counterparts. Thus, there is a need for a method of controlling the expression of multiple transgenes in a cell.

**Figure 1.**
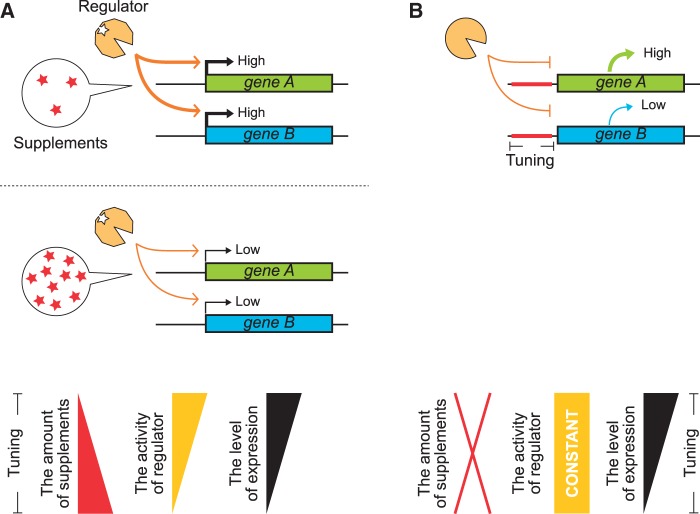
Schematic illustration of quantitative regulation of multiple transgenes. (**A**) Tuning of *trans*-acting regulators using supplement-responsive systems, such as a tetracycline-controlled transactivator (8). The concentration of a small molecule determines the activity of *trans*-acting regulatory proteins in the cells. As a result, multiple genes are expressed at similar levels in the same cell. (**B**) Tuning of *cis*-regulatory elements in each gene. In this case, the expression levels of multiple genes in the same cell are tuned simultaneously but independently under the regulator. The activity of the regulator remains constant in this system.

Thus far, especially in higher eukaryotes, quantitative regulation of protein expression from exogenous genes has largely depended on transcription factors that respond to supplemented small molecules, such as tetracycline (1,2,4) (Figure 1A). The concentration of the supplement determines the activity of the factors (8,9), each of which can evenly adjust the transcription levels of multiple target genes in a cell (Figure 1A). In one study, doxycycline modulated the activities of two transcriptional regulators to enable reversible control of two target genes (10). In another instance, several *trans*-acting regulatory factors were used to regulate multiple transgenes under the control of a single stimulus (11,12). However, although only one small molecule stimulant was used in these systems, multiple *trans*-acting regulatory proteins or RNAs were required to control multiple transgenes in these cases. Simultaneous and specific regulation of multiple transgenes with a single regulatory protein (Figure 1B) could ameliorate the acute shortage of well-characterized regulatory proteins (especially in mammalian systems) and minimize the risk of cross-talk among transgenes and intrinsic gene regulatory networks.

It has been previously reported that libraries of synthetic promoters with a range of activity have been developed as *cis* transcriptional regulatory elements (13,14), enabling independent control of multiple transgenes. However, the activity using these synthetic promoter libraries was constitutively determined by endogenous transcription factors, resulting in difficulty in controlling expression of transgenes with an internal or external stimulus. Moreover, it is difficult to rationally determine expression levels of multiple genes with these library-based screening approaches.

Recently, in addition to transcriptional regulation, means for fine-tuning translational efficiency have gained in importance. For example, programmable mammalian biocomputers have been developed using both transcriptional and translational regulatory components (15). So far, few methods have been developed for translational regulation of genes of interest in mammalian cells (16,17). We have reported a translational OFF switch that strongly represses the translation of a target mRNA designed to contain the binding motif [termed the kink-turn RNA motif (Kt)] of an archaeal ribosomal protein, L7Ae (18,19). However, the binary switch-like behavior of the system has limited its use for quantitative regulation. In a previous report, we took the first step toward a quantitative system by demonstrating tunable feedback regulation by a set of modified L7Ae proteins with engineered binding properties (20). In this study, we aimed to develop complementary approaches for quantitatively tuning translation of distinct mRNAs by an RNA-binding protein. We describe two independent methods for finely tuning protein expression without modification of the RNA–protein interaction. It has been reported that protein-binding RNA motifs inserted close to the 5′-end of an mRNA effectively repress translation in the presence of their corresponding proteins (21–23). We examined the dependence of translational repression in our system on the placement of the inserted RNA motif, and we found that the efficiency of translation adjusts continuously in a manner that correlates well with the distance between the binding motif and the 5′ terminus of the mRNA. Tandem insertion of multiple RNA motifs into one mRNA further modifies the inhibition. Combining these two mechanisms allows us to fine-tune the translational efficiency of the target mRNAs over a wide range. Simultaneous and specific control of two different transgenes was rationally designed and experimentally demonstrated by engineering each *cis*-regulatory 5′-UTR. Tuning *cis*-regulatory elements instead of *trans*-acting effectors enables simultaneous, specific and quantitative control of target genes (Figure 1B).

## MATERIALS AND METHODS

### Plasmid constructions

Reporter plasmids that express enhanced green fluorescent protein (EGFP) or enhanced cyan fluorescent protein (ECFP) following engineered 5′-UTRs were derived from pKt-EGFP and pdKt-EGFP, previously described as p1-box C/D-EGFP and p1-box C/D mut-EGFP (18), respectively. The region encoding EGFP was replaced by ECFP to generate pKt-ECFP and pdKt-ECFP, referred to as 32 nt in the length of the 5′-UTR. Spacer sequences were amplified by polymerase chain reaction (PCR) from *LacZ* gene using primer sets (5′-CCCGGGATCCGATCCCGTCGTTTTACAAC-3′/5′-AGATCTACCGGTCAGGCTGCGCAAC-3′ and 5′-GGATCCGCTAGCGATACACCGCATC-3′/5′-ACTAGTAGATCTCAATGGCAGATCCCAG-3′), digested and ligated between the BamHI–AgeI sites and the NheI–BglII sites of pKt-EGFP to create plasmids pKt-Sp-EGFP and pSp-Kt-EGFP, respectively. Similarly, pdKt-Sp-EGFP and pSp-dKt-EGFP were generated from pdKt-EGFP.

pKt-ECFP and pdKt-ECFP were digested with NheI and BamHI, blunted by Klenow fragment (Takara Bio, Otsu, Japan) and self-ligated to create the shortest 5′-UTR (18 nt). The longest spacer sequence (320 nt) was generated by concatenation of the two spacer fragments described earlier in the text and inserted between the NheI–BglII sites of pKt-ECFP and pdKt-ECFP. The remaining series of spacers were amplified with proper primer sets and inserted into the 5′-UTRs of the reporter plasmids in a similar manner. All the sequences of the 5′-UTRs from the reporter plasmids containing Kt are available in Supplementary Table S1.

Pairs of oligonucleotides [Kl (20): 5′-CATGGGATCCGGGTGTGAACGGTGATCACCCGA-3′/5′-GATCTCGGGTGATCACCGTTCACACCCGGATCC-3′, Kl2: 5′-CATGGGATCCGGACGTACGTGTGAACGGTGATCACGTACGCCGA-3′/5′-GATCTCGGCGTACGTGATCACCGTTCACACGTACGTCCGGATCC-3′, MS2SL (24): 5′-CATGGGATCCGGTGAGGATCACCCATCGA-3′/5′-GATCTCGTTGGGTGTTCCTCTCCGGATCC-3′] were annealed and cloned into a cloning vector. Fr15 (25) was amplified by PCR using a primer set (5′-GCTAATCCATGGGATCCTCGGTCGAAAGACTTGAGGGC-3′/5′-CCCAGATCTCGTCAGGTGCAGGCCAGAC-3′) from DNA templates (5′-GGGATGTCAGGTGCAGGCCAGACCGAAGTCCTCTCCTGCCCTCAAGTCTTTCGACCATCCCTATAGTGAGTCGTATTAGC-3′), digested by NcoI and BglII and cloned into the vector as well. Each RNA motif was concatenated using BamHI at the 5′-end and BglII at the 3′-end of the cloning vector. Then single or multiple RNA motifs were extracted from the cloning vector by BamHI and BglII digestion and inserted between the BglII–BamHI sites of the vectors that contain Kt at the 67th, 120th and 164th nt from the 5′-end. The same fragments of RNA motifs were also inserted into the BamHI site of pKt-ECFP and placed at the 18th nt from the end by blunting and self-ligation as described earlier in the text. The sequences of these 5′-UTRs are also available in Supplementary Table S2.

Trigger plasmids express RNA-binding proteins, which are fused to a One-STrEP-tag (IBA, Göttingen, Germany) at the N-terminus and to a myc-tag and His-tag at the C-terminus, with IRES-driven DsRed-Express under the control of a cytomegalovirus (CMV) promoter. In brief, the RNA-binding proteins were cloned into pcDNA5/FRT/TO (Invitrogen, Carlsbad, CA, USA) containing an expression cassette from pIRES2-DsRed-Express (Clontech Laboratories, Mountain View, CA, USA).

pIRES2-DsRed-Express was digested with BamHI and NotI, and the fragment containing the IRES2-driven DsRed-Express expression cassette was cloned into the BamHI–NotI site of pcDNA5/FRT/TO (Invitrogen). The HindIII-digested fragment from p4LambdaN22-3mEGFP-M9 (26) was inserted into the resulting expression vector. Then four-times repeated λ N22 peptide was replaced by the RNA-binding proteins that were amplified by PCR and fused to the peptide tags. The open reading frames of *Archaeoglobus fulgidus* L7Ae, bacteriophage MS2 coat protein and *Bacillus stearothermophilus* S15 were amplified using primer sets (5′-GAATCCATGGGATCCATGTACGTGAGATTTGAGGTTC-3′/5′-CACCAGATCTCTTCTGAAGGCCTTTAATCTTCTC-3′, 5′-CACCATGGGATCCGCTTCTAACTTTACTCAGTTCGTTCTC-3′/5′-TATGAGATCTGTAGATGCCGGAGTTGGC-3′ and 5′-GACACCATGGGATCCGCATTGACGCAAGAGCG-3′/5′-TATGAGATCTTCGACGTAATCCAAGTTTCTCAAC-3′) from plasmids pL7Ae (18), MS2-EGFP (27) and a newly synthesized plasmid based on the past study (28), respectively.

### Cell culture and transfections

**Figure 2.**
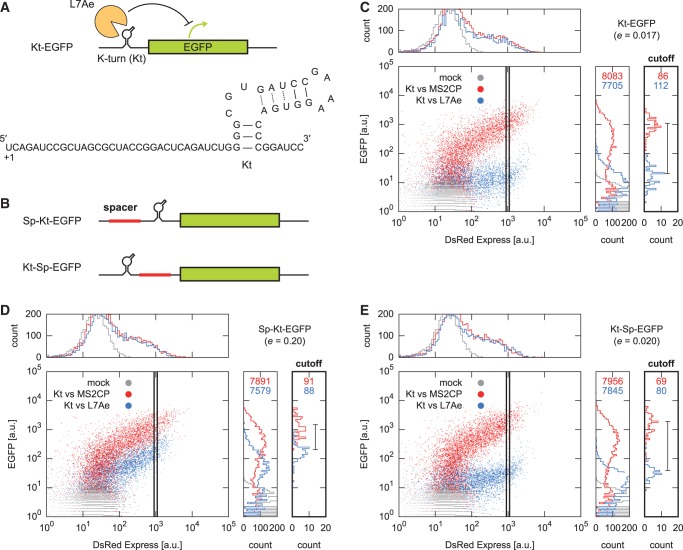
Insertion of a spacer between the 5′ terminus and the K-turn (Kt) diminished the reactivity of the switch. (**A**) The sequence and the secondary structure of the original translational OFF switch containing the Kt motif (Kt-EGFP) described as p1-box C/D-EGFP in a previous study (18). The transcriptional start site of the CMV promoter is indicated as +1. A dotted line shows non-canonical base pairing. (**B**) Schematic illustration of switches. A spacer sequence (red box) was inserted into the 5′-UTR upstream (Sp-Kt-EGFP) or downstream (Kt-Sp-EGFP) of Kt. (**C–E**) Plots from a flow cytometric analysis. HeLa cells were transfected with either pKt-EGFP (C), pSp-Kt-EGFP (D) or pKt-Sp-EGFP (E) in addition to a plasmid expressing MS2 coat protein (red, MS2CP) and L7Ae (blue). Mock (gray) indicates untransfected cells. DsRed-Express was synthesized from the same vector as the RNA-binding proteins. Upper and right panels show histograms of the fluorescence of the DsRed-Express and EGFP, respectively. Black boxes in plots indicate cut-off used to calculate the translational efficiency (*e*) of the switch. The right most panels show histograms in the cut-off, and the bar in the panels indicates the change of the average EGFP fluorescence. The number of events in plots is shown in the right panels. Representative results are shown out of three independent experiments, except translational efficiencies, which were the average of the three experiments.

**Figure 3.**
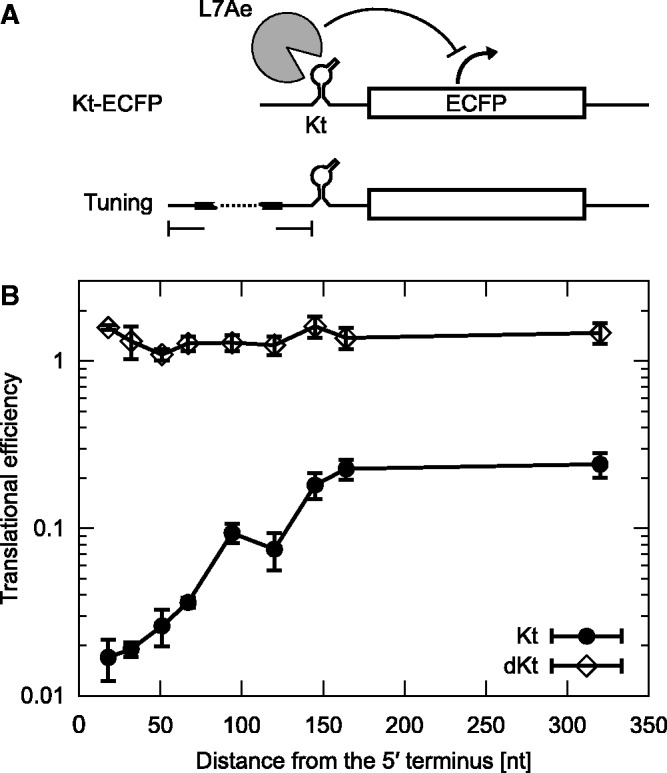
Correlation between the efficiency of translation and the distance between the 5′-end of the mRNA and Kt. (**A**) Construction of engineered mRNAs, which were tuned by altering the distance of the motif from the 5′-end. ECFP was used in this study as a reporter. Binding of L7Ae to Kt in the 5′-UTR represses the production of the reporter protein. A black box with a dotted line indicates spacer sequences with various lengths. (**B**) Translational efficiency of the engineered mRNAs with Kt (black circles) or defective Kt (dKt, open diamonds) as a function of the length between the 5′ terminus and the motif. Translational efficiency was defined as a ratio of the ECFP fluorescence from L7Ae-expressing cells divided by that from cells expressing non-cognate MS2 coat protein. Plots of flow cytometric analyses are available in Supplementary Figure S2. The average and standard deviation of three independent experiments are shown.

**Figure 4.**
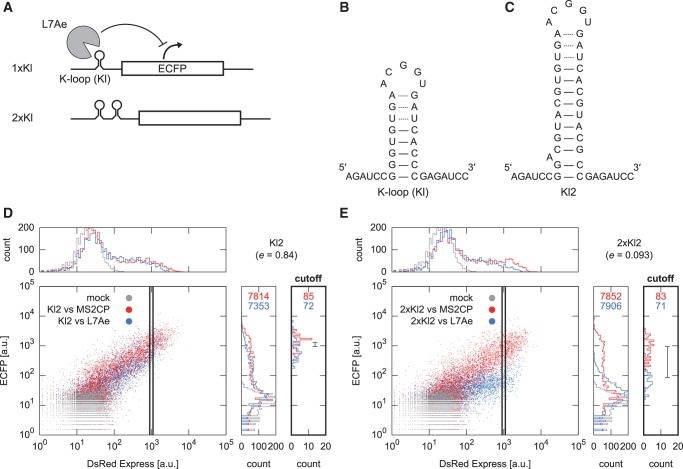
Inserting multiple K-loop motifs enhanced the reactivity of the switch. (**A**) Construction of engineered mRNAs, which were tuned by altering the number of motifs. (**B**) Secondary structure of K-loop (Kl) used in this study. Non-canonical base pairing is shown in dotted lines. (**C**) Modified K-loop (Kl2) used only in this experiment. (**D** and **E**) Results of the flow cytometric analysis of cells expressing switches containing one Kl2 (D) or two tandemly repeated Kl2 (2× Kl2; E) in the presence of MS2 coat protein (red) or L7Ae (blue). All the data are shown as in Figure 2. Representative results are shown out of the three independent experiments, except translational efficiencies, which were the average of the three experiments.

**Figure 5.**
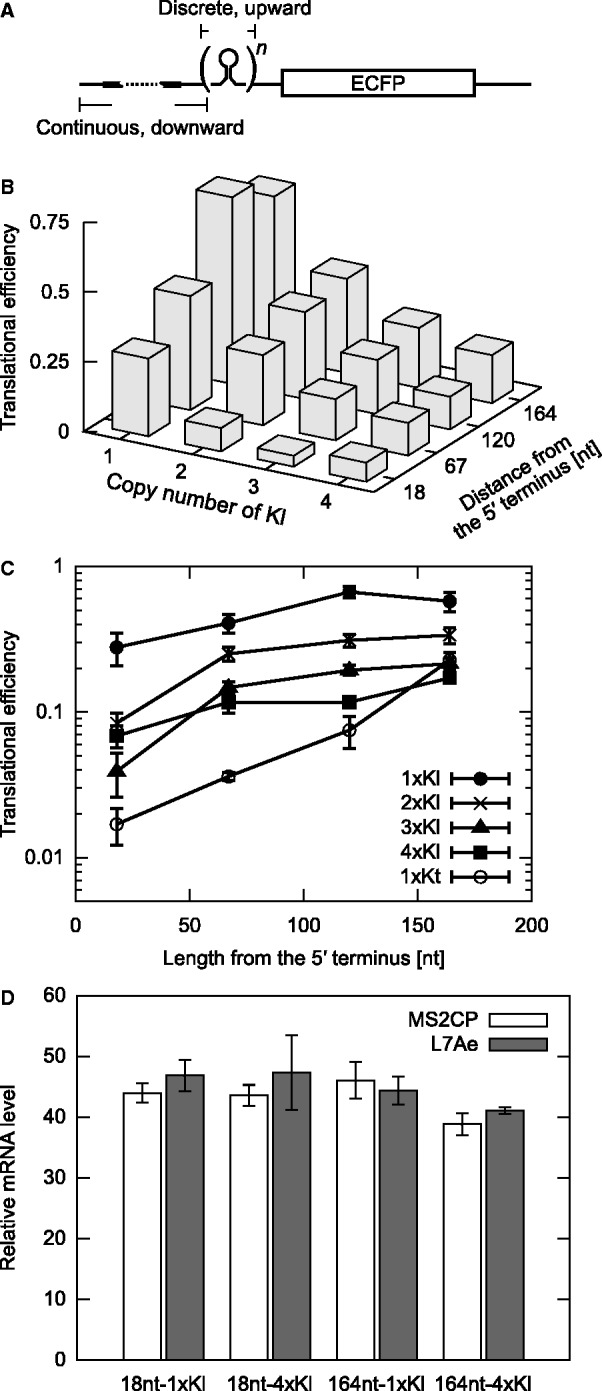
2D tuning of protein expression from engineered mRNAs. (**A**) Strategy for engineering mRNAs with a 2D approach (altering both the position and the number of motifs). (**B**) Translational efficiency of 2D-tuned mRNAs as a function of the distance from the 5′ terminus and the number of K-loop (Kl) motifs. The average of three independent experiments is shown. (**C**) Plots of translational efficiencies presented in B. One (circle), two (cross), three (triangle) and four (square) copies of Kl were located at various distances from the 5′ terminus. A single-Kt-motif case (open circle, shown in Figure 3B) is also presented for comparison. The average and standard deviation from the three independent experiments are shown. (**D**) Intracellular levels of mRNAs with engineered 5′-UTRs. The amount of indicated reporter mRNAs in the presence of MS2 coat protein (MS2CP, open box) or L7Ae (filled box) was measured by quantitative reverse transcriptase–PCR and normalized using a neomycin-resistance gene expressed from the same vector as the reporter. The average and standard deviation of triplicate measurements are shown.

**Figure 6.**
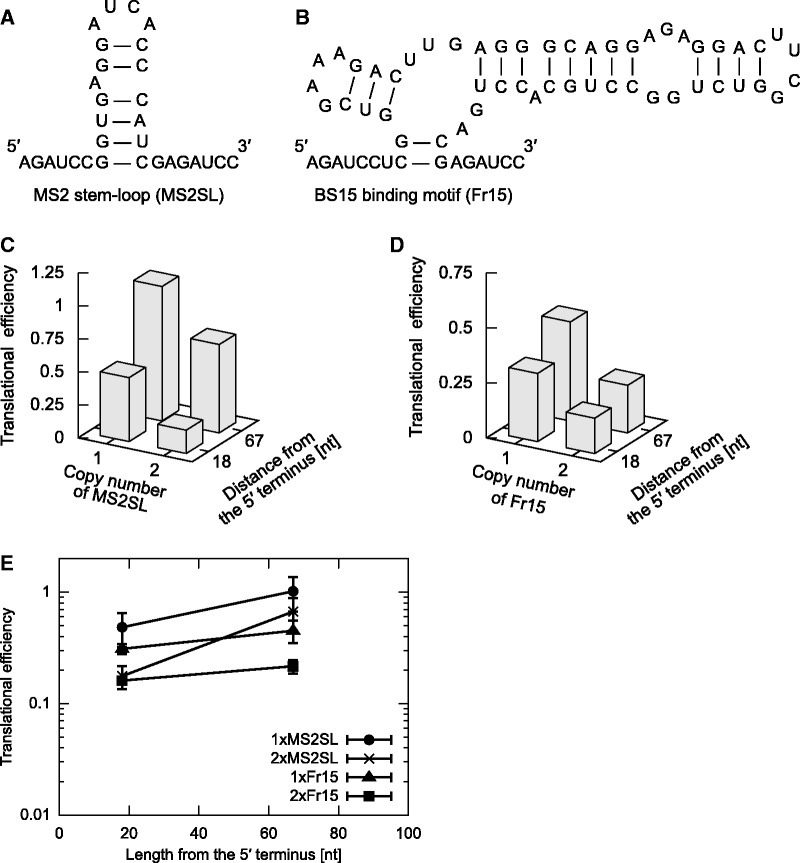
2D tuning of the OFF switches triggered by bacteriophage MS2 coat protein or ribosomal protein S15 from *B. stearothermophilus*. (**A** and **B**) Secondary structures of the RNA motif used in this study. MS2 stem–loop (MS2SL; A) and the fragment to which S15 binds (Fr15; B) were inserted into the switch instead of K-loop. (**C** and **D**) Translational efficiency of 2D-tuned switches containing MS2SL (C) and Fr15 (D). The averages of the three independent experiments are shown. (**E**) Plots of translational efficiencies presented in C and D. One (circle) and two (cross) copies of MS2SL and one (triangle) and two (square) copies of Fr15 were located at indicated distances from the 5′ terminus. The average and standard deviation from the three independent experiments are shown.

**Figure 7.**
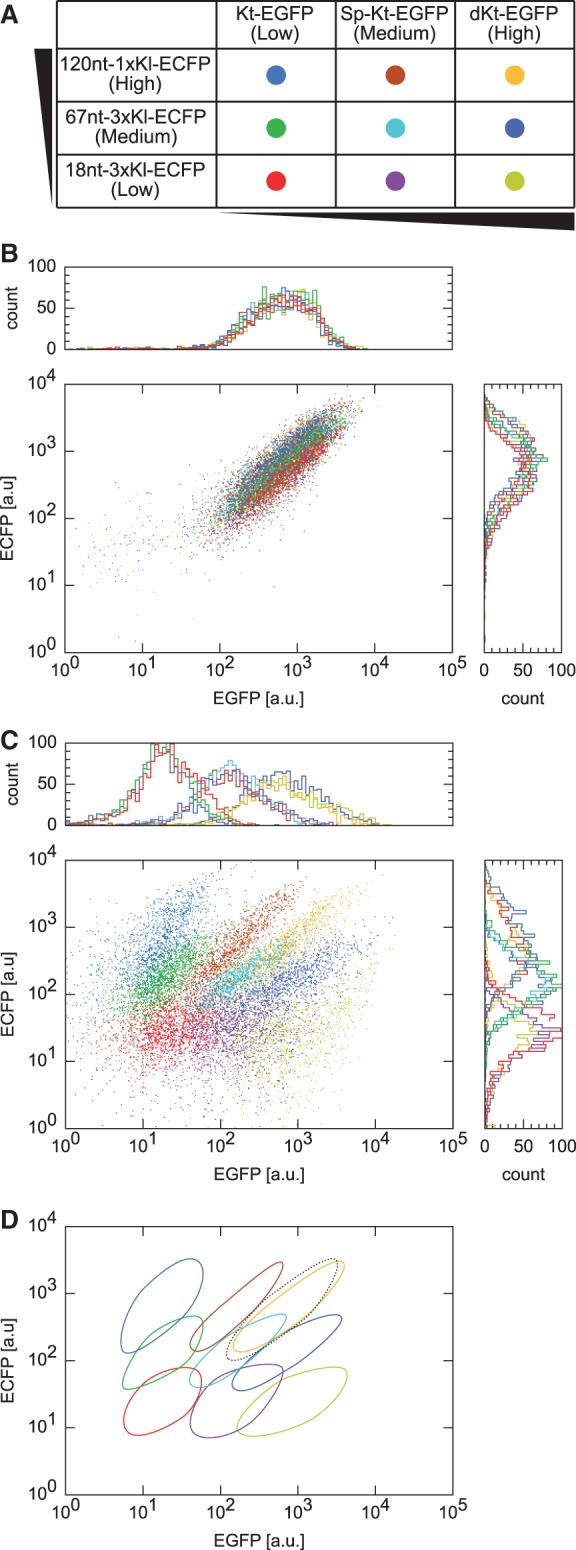
Simultaneous control of two independently tuned output proteins. (**A**) Pairs of tuned constructs were co-transfected into cells. Kt-EGFP and 18 nt-3× Kl-ECFP were tuned to low-protein expression levels, dKt-EGFP and 120 nt-1× Kl-ECFP were tuned for high expression and Sp-Kt-EGFP and 67 nt-3× Kl-ECFP were tuned for medium expression. (**B** and **C**) Flow cytometric analysis of cells expressing two tuned mRNAs in the absence (B) and in the presence (C) of L7Ae. Plots are shown in the color scheme listed in A. Representative data out of three independent experiments are shown. Plots of EGFP and ECFP as a function of DsRed-Express are available in Supplementary Figure S3. (**D**) Schematic representation of the plots shown in C. The oval with the black dotted outline indicates all the pairs shown in B.

HeLa cells were cultured at 37°C and 5% CO_2_ in Dulbecco’s modified Eagle’s medium (GIBCO, Carlsbad, CA, USA) containing 10% fetal bovine serum (Nichirei Biosciences, Tokyo, Japan) and 1% antibiotic–antimycotic solution (Sigma-Aldrich, St Louis, MO, USA). In all, 5 × 10^4^ cells were seeded in 24-well plates, and after 24 h, 70–90% confluent cells were transiently transfected with plasmids using 1 µl of Lipofectamine 2000 (Invitrogen) following the manufacturer’s instructions. In the double-transfection experiments (Figures 2–6), 0.1 µg of a reporter plasmid and 0.5 µg of a trigger plasmid were transfected into cells. In the triple-transfection experiment (Figure 7), 0.1 µg each of two reporter plasmids and 0.3 µg of a trigger protein plasmid were used. Media were changed 4 h after transfection.

### Flow cytometric measurement

Twenty-four hours after transfection, cells were washed with phosphate-buffered saline and incubated in 100 µl of 0.25% Trypsin–ethylenediaminetetraacetic acid (GIBCO) for 2 min at 37°C. After addition of 100 µl of the medium, cells were passed through a 35-µm strainer (BD Biosciences, San Jose, CA, USA) and then analyzed with a FACS Aria (BD Biosciences). A 408-nm semiconductor laser for excitation and a 450/40-nm filter for emission were used to measure the fluorescence of ECFP. A 488-nm semiconductor laser and 530/30 and 695/40 nm filters were used for EGFP and DsRed-Express, respectively.

### Flow cytometric analysis

Dead cells were gated out by forward and side scatter properties. Translational efficiency (*e*) is defined in this study as the ratio of an average of the EGFP or ECFP fluorescence in the presence of cognate RNA-binding protein divided by that of non-cognate RNA-binding protein from cells expressing an arbitrarily chosen 1000 ± 100 a.u. of DsRed-Express. Different cut-off of DsRed-Express (500 ± 50 a.u.) did not change the translational efficiencies throughout the experiments performed in Figures 2–7 (Supplementary Figure S1). In double-transfection experiments using the EGFP and DsRed-Express (Figure 2), and the triple-transfection experiments (Figure 7), the levels of fluorescence were compensated in a similar manner as described in the previous study (29). Coefficients of a spectral (spillover) matrix were determined from a data set of cells expressing EGFP, ECFP or DsRed-Express by the least-square method. Then, signal estimates for the reporter proteins were obtained by applying the compensation matrix, which was the inverse of the spectral matrix, to the observed data. In the triple-transfection experiment (Figure 7), untransfected cells were gated out based on the level of DsRed-Express (<100 a.u.). All the experiments were repeated three times, and the average and the standard deviation are presented.

### Isolation of total RNA and complementary DNA (cDNA) synthesis

Twenty-four hours after transfection, cells were washed with chilled phosphate-buffered saline. Total RNA was isolated using the RNAqueous-4PCR Kit (Ambion, Carlsbad, CA, USA), following the manufacturer’s instructions. In all, 350 µl of lysis/binding solution was used, and RNA was eluted in 50 µl of Milli-Q water twice. Contaminating DNA was removed using the TURBO DNA-free Kit (Ambion), following the manufacturer’s instructions. cDNA was synthesized from 200 ng of the extracted total RNA using High-Capacity cDNA Reverse Transcription Kits (Applied Biosystems, Carlsbad, CA, USA). Resulting cDNA solutions were diluted 10-fold, and a 5-µl aliquot was subjected to quantitative PCR analysis.

### Quantitative PCR analysis

Quantitative PCR analysis was carried out using LightCycler 480 SYBR Green I Master (Roche, Basel, Switzerland) and LightCycler 480 instruments (Roche). The reaction solutions contained primer sets (5′-GAAGCGCGATCACATGGT-3′/5′-CCATGCCGAGAGTGATCC-3′) or (5′-GGCTACCCGTGATATTGCTG-3′/5′-GCGATACCGTAAAGCACGA-3′) at a final concentration of 500 nM to measure the mRNA levels of reporter ECFP or neomycin-resistant gene, respectively. A series of 10-fold dilutions of the reporter plasmid (50 fg to 5 ng) was used as a standard. Relative amount of the reporter ECFP mRNA was determined as a ratio to that of neomycin-resistance gene that was expressed from the same plasmid vector as the reporter. The average and standard deviation from the triplicate experiments were presented.

## RESULTS

### Quantitative regulation is correlated with the distance of the RNA motif from the 5′-end of the mRNA

We initially investigated the effect of the position of the protein-binding RNA motif in the 5′-UTR of the mRNA. A spacer sequence of ∼150 nt containing no AUG codon was amplified from the *lacZ* gene and inserted upstream or downstream of Kt in our original translational OFF switch construct (Kt-EGFP; Figure 2A) regulating EGFP expression (Figure 2B and Supplementary Table S1). Each of the resulting plasmids was co-transfected into HeLa cells with a plasmid expressing either a cognate or a non-cognate RNA-binding protein (L7Ae or bacteriophage MS2 coat protein, respectively). Twenty-four hours after transfection, the level of EGFP fluorescence was measured by a flow cytometer. Insertion of the spacer sequence between the 5′ terminus and Kt (Sp-Kt-EGFP) resulted in weakened translational repression (Figure 2C and D), whereas insertion of the spacer between Kt and the initiation codon (Kt-Sp-EGFP) gave no such effect (Figure 2C and E). This result confirms that the proximity of Kt to the 5′-end of the mRNA is important for translational repression in our system.

In this study, we define translational efficiency (*e*) as the ratio of reporter protein fluorescence from cells expressing cognate RNA-binding proteins to that from cells expressing non-cognate ones. In the case of co-transfection of two plasmids in cultured mammalian cells, although the total plasmid uptake varies widely, causing a broad distribution of protein production among the cells (30), the concentration ratio between the incorporated plasmids in the transfected cells seems constant. To normalize variations in transfection efficiency, we chose cells for analysis according to the fluorescence intensity (1000 ± 100 a.u.) of DsRed-Express, which was synthesized from an internal ribosome entry site on the mRNA encoding the RNA-binding proteins (Figure 2C–E, black boxes in plots). This range was chosen because the fluoresce intensity of cells with lower DsRed-Express was more sensitive to noise from autofluorescence. The translational efficiency values were similar among cells expressing DsRed-Express from 500 to 1000 a.u. (Supplementary Figure S1). We determined the translational efficiency of the Kt-EGFP and Sp-Kt-EGFP constructs. Insertion of the spacer upstream of Kt increased the translational efficiency from 0.017 (Kt-EGFP; Figure 2C) to 0.20 (Sp-Kt-EGFP; Figure 2D).

To enable the design of mRNAs that express proteins at any desired level, we more closely examined the relationship between the efficiency of translation and the distance between the 5′-end of the mRNA and Kt. We constructed a series of reporter mRNAs with Kt located at various sites between the 18th and 320th nt from the 5′ terminus (Figure 3A and Supplementary Table S1) and evaluated the effect on protein synthesis. To avoid fluorescence spill over, we replaced EGFP with ECFP in the following experiments. In the presence of the regulatory protein L7Ae, the translational efficiency correlates well with the distance of Kt from the 5′-end (Kt; Figure 3B). When Kt was replaced with a defective Kt (dKt) to which L7Ae cannot bind *in vitro*, the protein expression levels were almost constant irrespective of the presence of L7Ae (dKt; Figure 3B). The results indicate that the binding between the mRNA and L7Ae, and not the distance between the open reading frame and the 5′-end of the mRNA, is responsible for the translational regulation. Interestingly, the translational efficiency continuously increased with the length of the spacer despite the constant expression level of L7Ae (Figure 3B and Supplementary Figure S2), indicating that translation can be tuned quantitatively by adjusting the distance between the RNA motif and the 5′ terminus of the mRNA.

### Two-dimensional approach to tune translational efficiency

The translational efficiency under repression by Kt and L7Ae ranged between 0.017 and 0.24, reaching a plateau when Kt was positioned beyond the 164th nt from the 5′-end of the mRNA (Figure 3B). To expand the versatility of the method by widening the tuning range, we designed 5′ UTRs that contained multiple protein-binding motifs (Figure 4A). Multiple RNA–protein interaction motifs have been used in series to enhance the binding between the RNA molecule and the corresponding protein. For example, tandem arrangements of protein-binding RNA motifs have been used as tools for visualizing target mRNA molecules in living cells (26), detecting RNA–protein interactions (31) or regulating the stability of mRNA (32). We first substituted Kt with a K-loop RNA motif (Kl) (33) that binds to L7Ae less tightly than does Kt (20) (Figure 4B and C). Translation from the mRNA containing Kl showed slight repression in the presence of L7Ae (Figure 4D). We next designed an OFF mRNA with two repeated Kls. The mRNA showed 9-fold stronger repression than an mRNA with a single Kl (Figure 4D and E), demonstrating that the translational efficiency can be tuned by altering the number of the RNA motif at the 5′-UTR.

We combined the two strategies described earlier in the text to allow fine-tuning of the OFF mRNA system over a wide dynamic range. Specifically, the continuous downward tuning provided by adjusting the distance of Kl from the 5′-end of the mRNA was used together with the upward discrete tuning resulting from using multiple Kls (Figure 5A). We constructed mRNAs containing one to four copies of Kl (1× to 4× Kl) in the 5′-UTR and placed the first motif at either the 18th, 67th, 120th or 164th nt from the 5′ terminus (Supplementary Table S2). Sixteen constructs were transiently expressed in HeLa cells and analyzed for their translational efficiency. As anticipated, the translational efficiency of the mRNAs increased or decreased as the distance of the first motif from the 5′-end of the mRNA or the number of RNA motifs increased, respectively (Figure 5B and C). The combination of the two tuning strategies successfully extended the range of the translational efficiencies of the mRNAs (from 0.039 to 0.67).

To see whether the reporter gene expression was regulated at the level of translation, the amount of designed mRNAs transiently expressed in the cells was analyzed by real-time quantitative PCR (Figure 5D). No significant difference was observed in the amount of the mRNAs with one or four repeated Kls, the first motif of which was located at the 18th or 164th nt from the 5′-end.

To assess the versatility of our method, we replaced Kl/L7Ae regulatory pair with another RNA–protein pair. We constructed two series of 2D-tuned mRNAs containing a MS2 stem–loop, to which MS2 coat proteins bind (24,27,31) (Figure 6A), or a binding motif for *Bacillus* ribosomal protein S15 (25,28) (Figure 6B). Translational efficiencies of these constructs in HeLa cells responded to tuning similarly to those containing Kl, although the sequence around the motif might weakly affect the translational efficiency (Figure 6C and D and Supplementary Table S2). Thus, it is conceivable that any desired RNA–protein interaction pair can be used for controlling translation. Taken together, our results show that 2D engineering of *cis*-regulatory motifs in the 5′-UTR of mRNAs (i.e. engineering both the position and the number of protein-binding motifs on the mRNA) enables quantitative tuning of transgene expressions over a wide range.

### Simultaneous control of two differently tuned mRNAs

We next tested whether a single, unmodulated effector protein is able to both simultaneously and specifically regulate multiple genes containing differently tuned *cis*-regulatory elements (Figure 1B). Two sets of differently tuned reporter plasmids were designed and transfected into cells in the presence and absence of the cognate RNA-binding protein. The first set encoded *EGFP* mRNA with a single Kt in the 5′-UTR (Figure 2), whereas the second set encoded *ECFP* mRNA with 2D arranged Kls in the 5′-UTR (Figure 5). Each set consisted of three differently tuned variants: low- (Kt-EGFP, 18 nt-3× Kl-ECFP), high- (dKt-EGFP, 120 nt-1× Kl-ECFP) and intermediate-expression constructs (Sp-Kt-EGFP, 67 nt-3× Kl-ECFP) (Figure 7A). In the absence of L7Ae, EGFP and ECFP were uniformly expressed in all nine cell populations regardless of their engineered 5′-UTR (Figure 7B). In contrast, in the presence of L7Ae, the expression levels of EGFP and ECFP varied depending on the nature of the constructs, resulting in nine different fluorescence profiles (Figure 7C and D).

Figure 7 shows that the protein output of each mRNA is unaffected by translation of the other. To further investigate whether our method can control translation of multiple genes independently, we calculated the translational efficiencies of EGFP (Table 1) and ECFP (Table2) constructs under the co-transfectional conditions (Supplementary Figure S3). Obtained values were consistent with those in the single-reporter experiments (Figures 2 and 5), indicating that our system can achieve simultaneous and specific translational regulation of the two distinct mRNAs using a unique regulatory protein, L7Ae.

**Table 1.** Translational efficiencies of EGFP constructs are constant across the experiments and independent of co-transfected reporters

	Co-transfected reporter			
	18 nt-3× Kl-ECFP	67 nt-3× Kl-ECFP	120 nt-1× Kl-ECFP	None
Kt-EGFP	0.026 ± 0.010	0.021 ± 0.0074	0.019 ± 0.0018	0.017 ± 0.0015
Sp-Kt-EGFP	0.37 ± 0.0075	0.24 ± 0.053	0.22 ± 0.029	0.20 ± 0.051
dKt-EGFP	1.8 ± 0.29	2.1 ± 0.27	1.5 ± 0.31	1.3 ± 0.91^a^

^a^Translational efficiency from the ECFP construct (32 nt-dKt) shown in Figure 3 and Supplementary Figure S2.

**Table 2.** Translational efficiencies of ECFP constructs are constant across the experiments and independent of co-transfected reporters

	Co-transfected reporter			
	Kt-EGFP	Sp-Kt-EGFP	dKt-EGFP	None
18 nt-3× Kl-ECFP	0.037 ± 0.0024	0.028 ± 0.0054	0.048 ± 0.020	0.039 ± 0.013
67 nt-3× Kl-ECFP	0.22 ± 0.056	0.21 ± 0.036	0.23 ± 0.034	0.15 ± 0.015
120 nt-1× Kl-ECFP	0.77 ± 0.085	0.70 ± 0.19	0.59 ± 0.17	0.67 ± 0.059

## DISCUSSION

We have developed a method for independently regulating protein production from distinct target mRNAs in mammalian cells. The translation from each mRNA can be controlled across a wide dynamic range with a regulatory RNA-binding protein. We found that the location of the protein-binding motif in the 5′-UTR determines the translational efficiency. Translational repression was maximized when the motif was located close to the 5′ terminus of mRNA, and gradually decreased to become constant when the motif was located away from the 5′-end (Figure 3). We believe that our mRNA design-based approach could be advantageous compared with library screening-based approaches because expression levels of multiple genes can be rationally and quantitatively tuned by engineering both the position and the number of protein-binding motifs on the mRNA in a predictable manner.

Interestingly, the translational efficiency of the constructs was not affected in the presence of another construct in a cell (Figure 7, Tables 1 and 2 and Supplementary Figure S3), indicating that L7Ae has no preference for RNA motifs located at any particular position on the mRNA. Together with our previous study, which showed the expression level of L7Ae correlates with the translational efficiency (19), these results suggest that the ratio of L7Ae to the total of all the Kt-containing mRNAs in a cell determines the efficiency. If the amount of L7Ae is indeed the limiting factor, it is conceivable that a large number of distinct mRNAs can be simultaneously and specifically controlled provided that a sufficient amount of L7Ae is present.

Translational regulation using a protein-binding site in the 5′-UTR has been shown in a yeast system (22), indicating that the method is applicable to a variety of eukaryotes and RNA-binding proteins. Indeed, we found that MS2 coat protein or *Bacillus* ribosomal protein S15 with their corresponding RNA motifs can be used as alternative pairs (Figure 6), demonstrating the versatility of our method. Accordingly, an endogenous RNA-binding protein expressed in a specific cell could be a trigger for the control of target transgene expression. It is also conceivable that a synthetic RNA aptamer for a specific protein can be adapted to our system (34). In addition, we expect that the method described in this study can be applied for a wide range of protein outputs (e.g. fluorescent reporter proteins and apoptosis regulator proteins) (Supplementary Table S3).

In prokaryotes, the level of translation can be modulated in *cis* with engineered ribosome-binding sites (35,36). To express target proteins at desired levels, the sequences of ribosome-binding sites have been rationally designed based on a biophysical model of translation initiation affected by RNA folding and hybridization, combined with an optimization algorithm (37). Furthermore, by using a multiplex genome engineering method, simultaneous optimization of the translational efficiency of 24 genes in the same metabolic pathway enabled a biosynthesis circuit in *Escherichia coli* to overproduce industrially important chemical compounds (38). However, alternative approaches are required to control translation in mammalian cells, which do not share the ribosome-binding sites used in prokaryotic cells. Our approach, which enables us to tune the translation of multiple genes specifically, may allow genetic circuits to be engineered and optimized in a eukaryotic system.

Our method for quantitatively tuning the expression of multiple transgenes can be used as an alternative tool for studying complex cellular processes and engineering cells for therapeutic uses. For example, it could be used to study the expression balance of multiple disease-related genes, or for controlling cellular phenotypes determined by threshold dose responses (1). The method can also be useful for controlling differentiation or self-renewal of stem cells, where the optimal stoichiometry of multiple transcription factors governs the fates of the cells (7). Direct injection of synthetic mRNAs into mammalian cells could serve as a powerful tool for gene therapy (39) and regenerative medicine (40) because transferred mRNAs do not integrate into the genome and pose no risk of causing cellular damage such as might lead to tumor formation. Thus, the combined use of RNA injection and our method could allow quantitative regulation of gene function in a cell.

We have previously shown that an L7Ae gene integrated into the genome under tetracycline-inducible transcriptional control regulates the translation of the anti-apoptotic protein Bcl-xL to allow control of mammalian cell fate (19). That result demonstrated that our translational regulation method can be used cooperatively with conventional transcriptional control systems because they exist as two completely different layers. In transcriptional control systems, transgenes are placed under specific promoters that determine the timing and location of their expression. In the translational control method described in this study, the translational efficiency of the target is determined by the RNA-binding protein produced in a cell. Accordingly, quantitative and simultaneous regulation of the production of multiple proteins should be temporally and spatially inducible in a cell containing a single regulatory protein (e.g. L7Ae)-coding gene under the control of a specific promoter.

A recent genome-wide analysis has revealed that post-transcriptional control of gene expression predominantly determines the amounts of proteins in mammalian cells (41). Moreover, it has been reported that translational control together with the localization of mRNAs significantly contributes to many important cellular processes, such as the establishment of polarity, asymmetric division, synaptic plasticity and memory consolidation (42). In the near future, our method may enable translational control to play the same leading role in synthetic regulatory systems as it does in natural ones.

## SUPPLEMENTARY DATA

Supplementary Data are available at NAR Online: Supplementary Tables 1–3 and Supplementary Figures 1–3.

## FUNDING

International Cooperative Research Project (ICORP), JST; Takeda Science Foundation (in part); New Energy and Industrial Technology Development Organization [NEDO, 09A02021a to H.S. in part]; Short-term Postdoctoral Fellowship from the Japan Society for the Promotion of Science (to J.A.S.). Funding for open access charge: Takeda Science Foundation.

*Conflict of interest statement.* None declared.

## Supplementary Material

Supplementary Data
