# Supplementary material for: Quantitative and simultaneous translational control of distinct mammalian mRNAs
Source: Nucleic Acids Res. 2013 May 18;41(13):e135. doi: 10.1093/nar/gkt347 (PMC3711428; doi:10.1093/nar/gkt347)
Supplement: Supplementary Data [file supp_41_13_e135__index.html]

Quantitative and simultaneous translational control of distinct mammalian mRNAs — Quantitative and simultaneous translational control of distinct mammalian mRNAs — Supplementary Data 

# Quantitative and simultaneous translational control of distinct mammalian mRNAs

## Supplementary Data

files

**Files in this Data Supplement:**

- Supplementary Data - pdf file
