# Supplementary material for: Quantitative and simultaneous translational control of distinct mammalian mRNAs
Source: Nucleic Acids Res. 2013 May 18;41(13):e135. doi: 10.1093/nar/gkt347 (PMC3711428; doi:10.1093/nar/gkt347)
Supplement: Supplementary Data [file supp_gkt347_nar-00865-met-g-2013-File003.pdf]

Supplementary Information

Quantitative and simultaneous translational control of distinct mammalian mRNAs

Kei Endo, James A. Stapleton, Karin Hayashi, Hirohide Saito and Tan Inoue

Table of Contents

Supplementary Figures S1–S3

Supplementary Figure S1	Values of translational efficiency from differently gated cell populations.
Supplementary Figure S2	Plots of a flow cytometric analysis presented in Figure 3B.
Supplementary Figure S3	Plots of a flow cytometric analysis presented in Figure 7.

Supplementary Tables S1–S3

Supplementary Table S1	Sequences of the 5' UTRs containing Kt.
Supplementary Table S2	Sequences of the 5' UTRs containing Kl, Kl2, MS2SL, or Fr15.
Supplementary Table S3	Verified combinations of an RNA motif for translation repression and an output protein.

Supplementary Figures

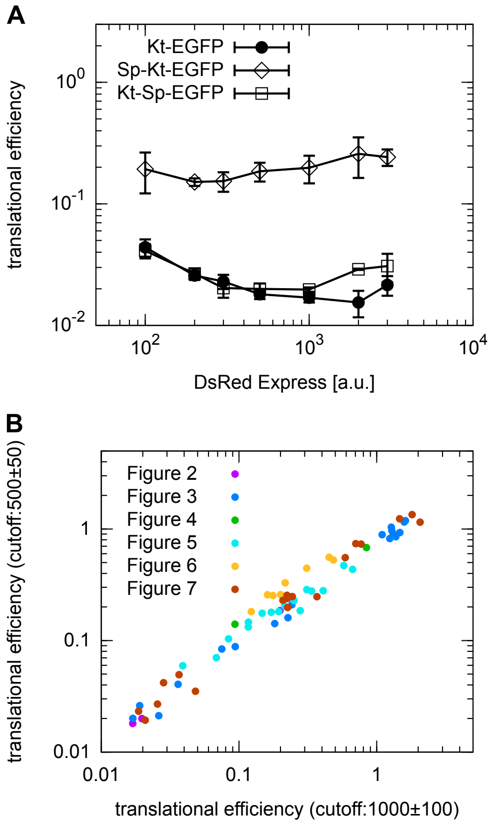

Supplementary Figure S1. Values of translational efficiency from differently gated cell populations. (**A**) A series of translational efficiencies from the same data as in Figure 2. Transfected HeLa cells were gated out based on the indicated intensity (± 10%) of DsRed-Express, and used for calculating translational efficiencies. The average and the standard deviation from the three independent experiments were shown. (**B**) Comparison of obtained translational efficiencies from 1000 ± 100 [a.u.] DsRed-Express cutoff and that from 500 ± 50 [a.u.] cutoff throughout the experiments (Figure 2–7). Correlation coefficient of these values was 0.97.

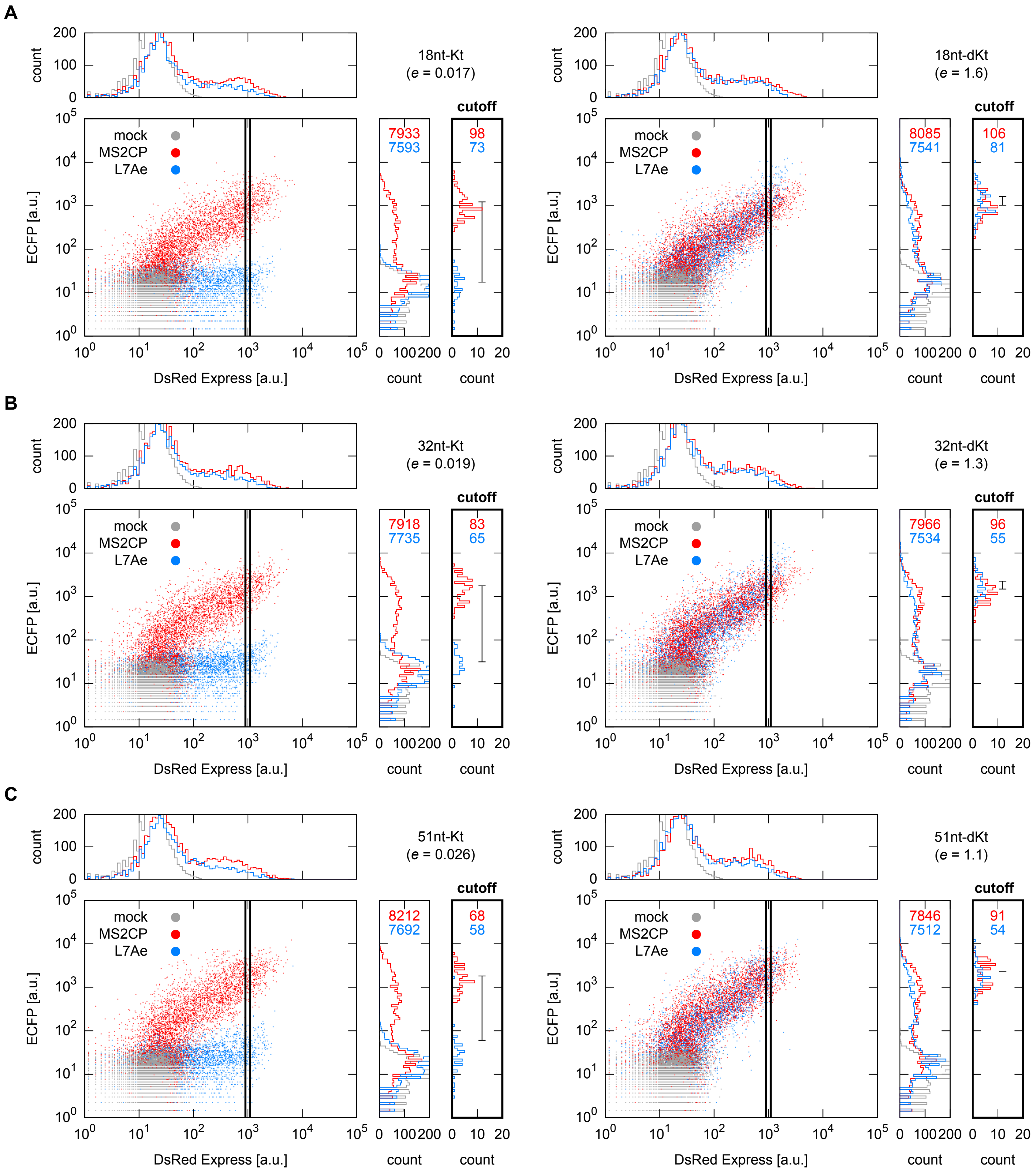

A
18nt-Kt
(e = 0.017)
18nt-dKt
(e = 1.6)
B
32nt-Kt
(e = 0.019)
32nt-dKt
(e = 1.3)
C
51nt-Kt
(e = 0.026)
51nt-dKt
(e = 1.1)
mock
MS2CP
L7Ae
cutoff
7933
7593
98
73
8085
7541
106
81
7918
7735
83
65
7966
7534
96
55
8212
7692
68
58
7846
7512
91
54
count
ECFP [a.u.]
DsRed Express [a.u.]

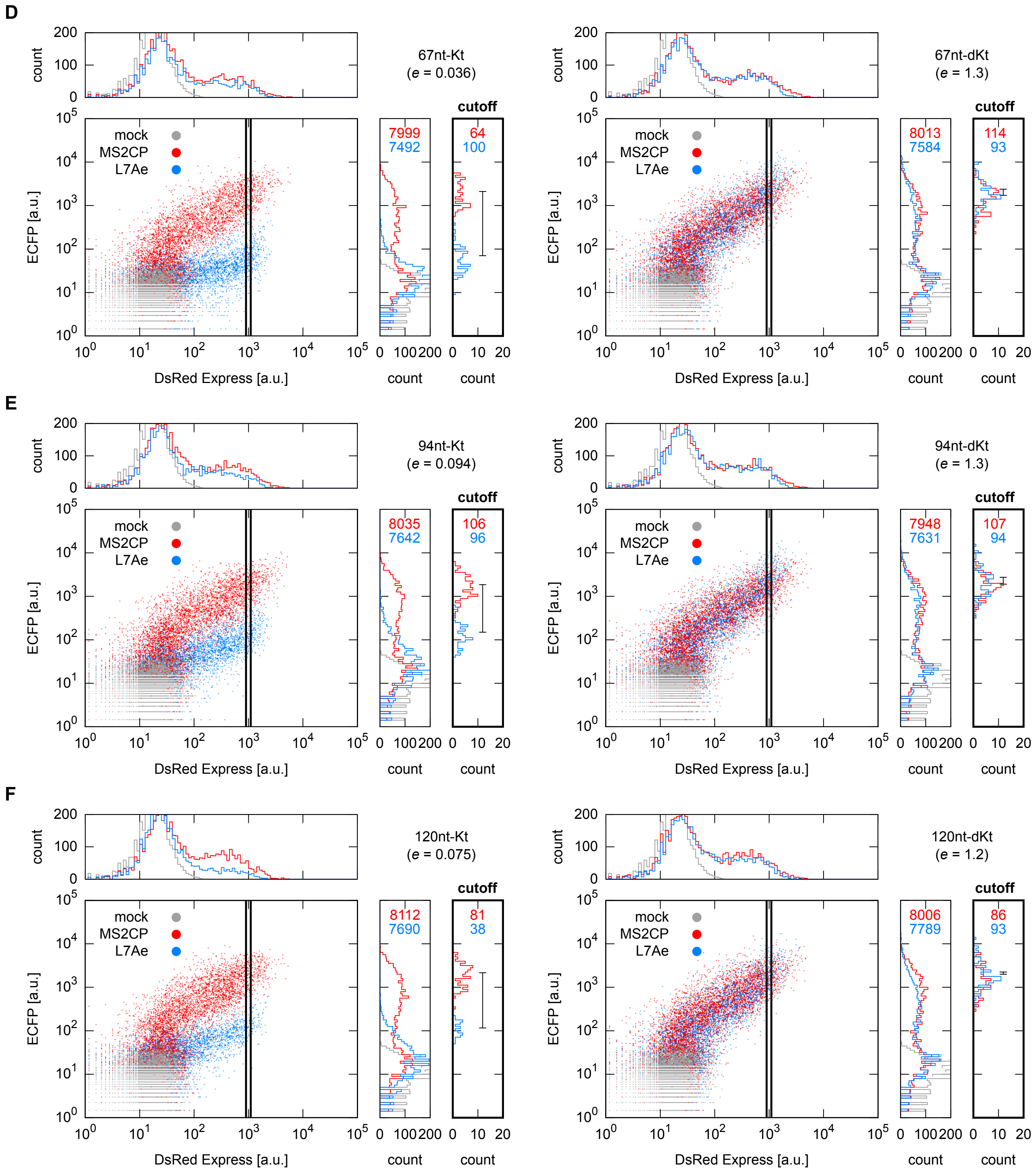
D
67nt-Kt
(e = 0.036)
67nt-dKt
(e = 1.3)
E
94nt-Kt
(e = 0.094)
94nt-dKt
(e = 1.3)
F
120nt-Kt
(e = 0.075)
120nt-dKt
(e = 1.2)
mock
MS2CP
L7Ae
cutoff
7999
7492
64
100
8013
7584
114
93
8035
7642
106
96
7948
7631
107
94
8112
7690
81
38
8006
7789
86
93
count
ECFP [a.u.]
DsRed Express [a.u.]
count
count

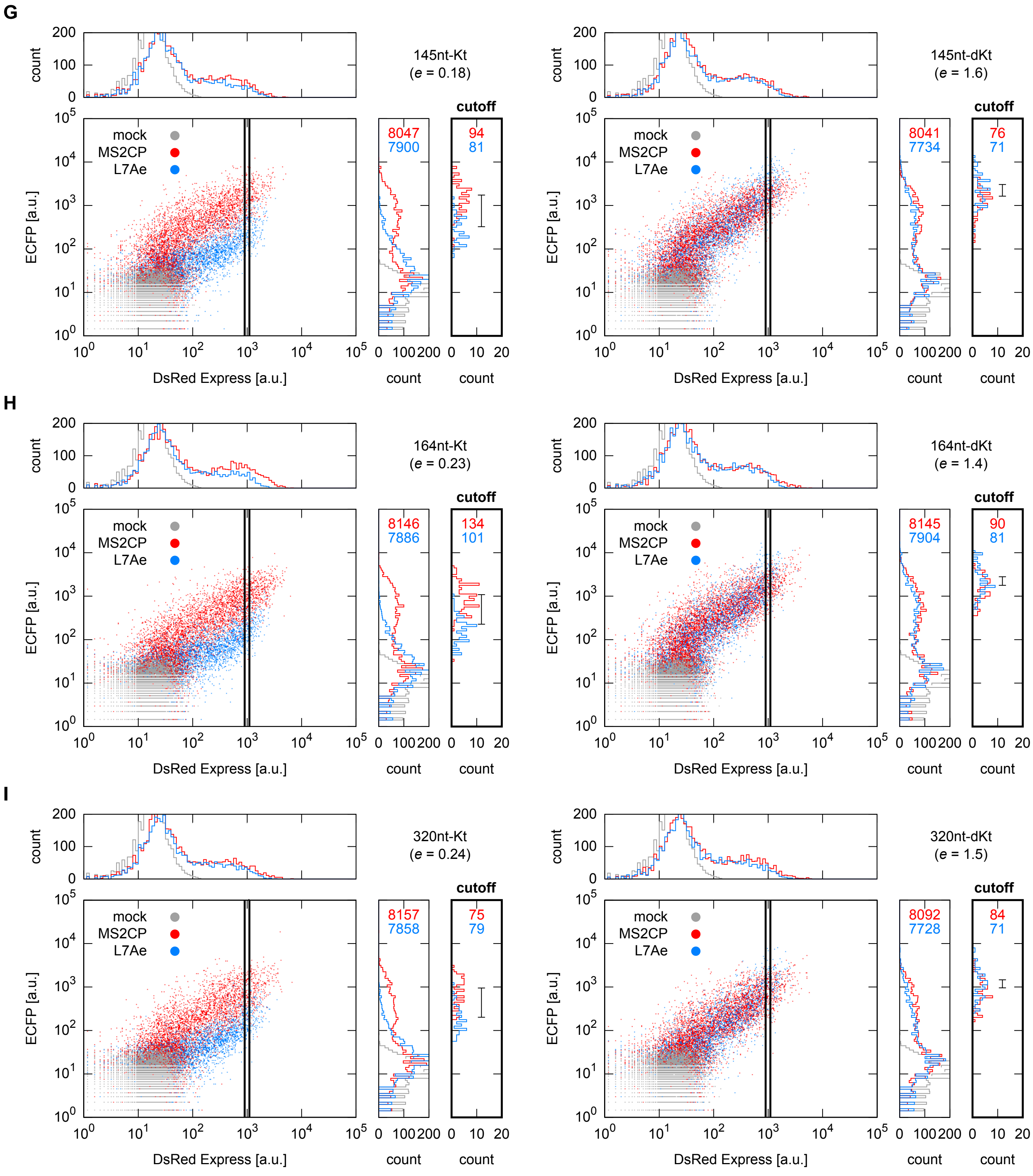
G
H
I
145nt-Kt
(e = 0.18)
145nt-dKt
(e = 1.6)
164nt-Kt
(e = 0.23)
164nt-dKt
(e = 1.4)
320nt-Kt
(e = 0.24)
320nt-dKt
(e = 1.5)
mock
MS2CP
L7Ae
cutoff
count
ECFP [a.u.]
DsRed Express [a.u.]
8047
7900
94
81
8041
7734
76
71
8146
7886
134
101
8145
7904
90
81
8157
7858
75
79
8092
7728
84
71

Supplementary Figure S2. Plots of a flow cytometric analysis presented in Figure 3B. Kt (left) or dKt (right) were located at 18th (**A**), 32nd (**B**), 51st (**C**), 67th (**D**), 94th (**E**), 120th (**F**), 145th (**G**), 164th (**H**) and 320th (**I**) nucleotide from the 5' terminus. All the data are shown as in Figure 2. Representative results out of three independent experiments are shown, except translational efficiencies that were the average of the three experiments.

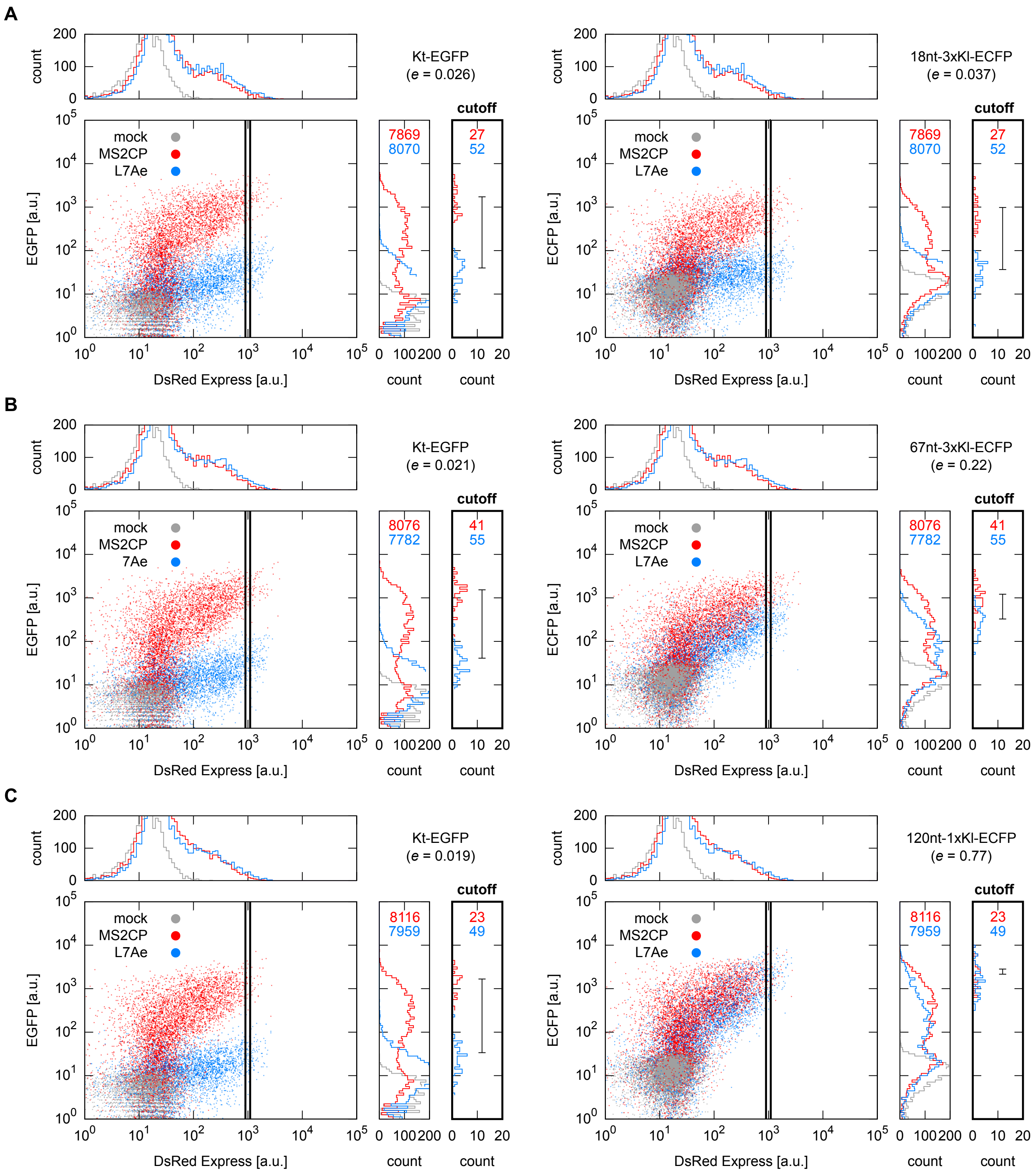
A
Kt-EGFP
(e = 0.026)
18nt-3xKI-ECFP
(e = 0.037)
cutoff
mock
MS2CP
L7Ae
7869
8070
27
52
EGFP [a.u.]
ECFP [a.u.]
DsRed Express [a.u.]
count
B
Kt-EGFP
(e = 0.021)
67nt-3xKI-ECFP
(e = 0.22)
8076
7782
41
55
7Ae
C
Kt-EGFP
(e = 0.019)
120nt-1xKI-ECFP
(e = 0.77)
8116
7959
23
49

D

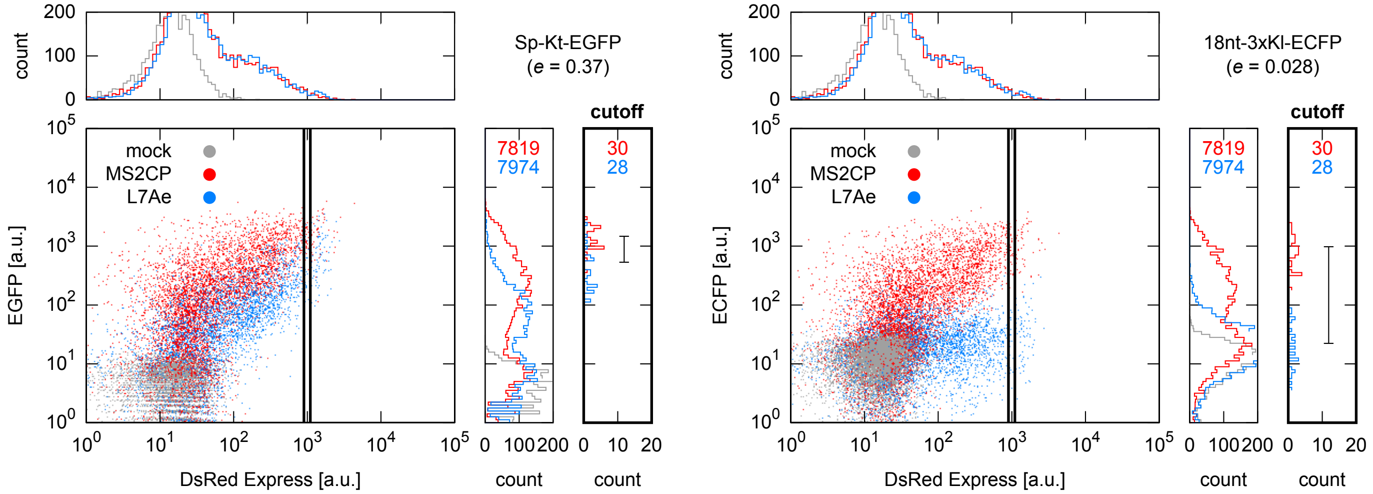
Sp-Kt-EGFP
(e = 0.37)
18nt-3xKl-ECFP
(e = 0.028)
mock
MS2CP
L7Ae
cutoff
7819
7974
30
28
EGFP [a.u.]
ECFP [a.u.]
DsRed Express [a.u.]
count

E

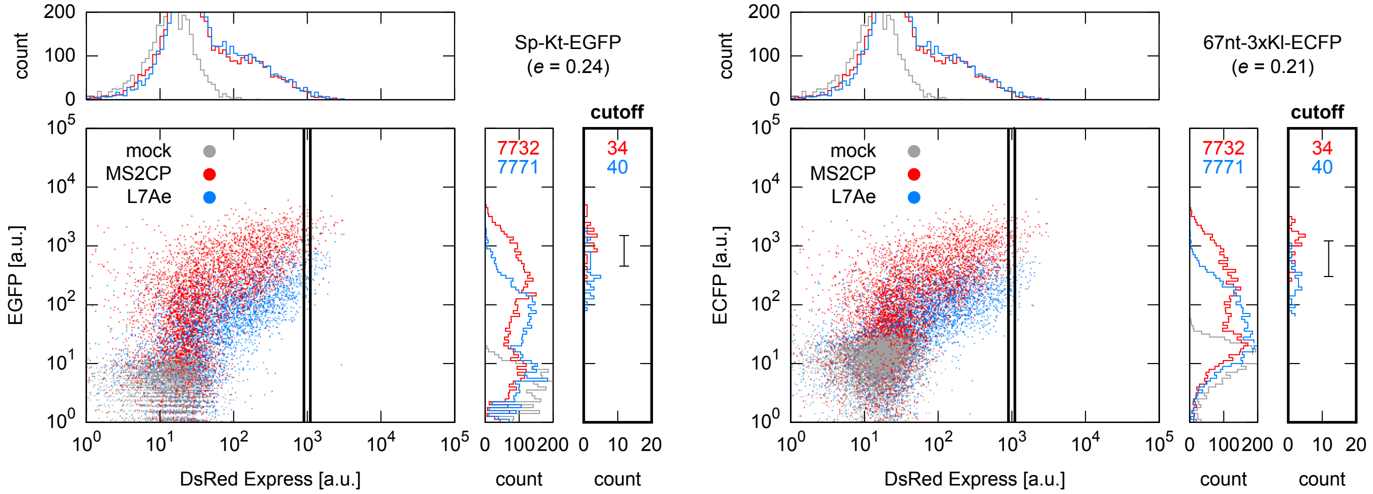
Sp-Kt-EGFP
(e = 0.24)
67nt-3xKl-ECFP
(e = 0.21)
mock
MS2CP
L7Ae
cutoff
7732
7771
34
40
EGFP [a.u.]
ECFP [a.u.]
DsRed Express [a.u.]
count

F

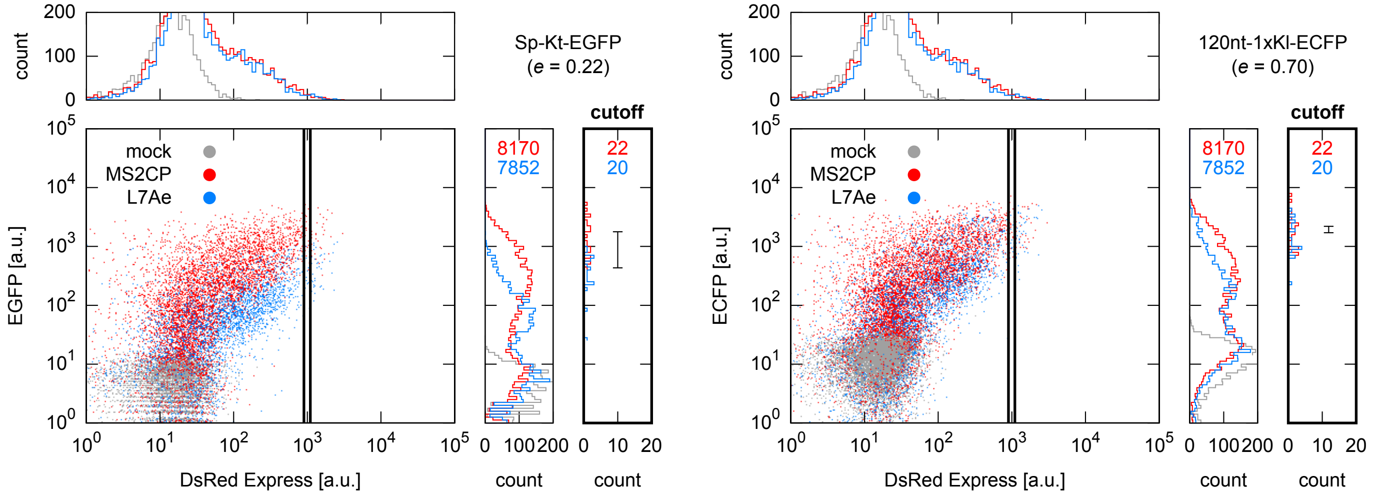
Sp-Kt-EGFP
(e = 0.22)
120nt-1xKl-ECFP
(e = 0.70)
mock
MS2CP
L7Ae
cutoff
8170
7852
22
20
EGFP [a.u.]
ECFP [a.u.]
DsRed Express [a.u.]
count

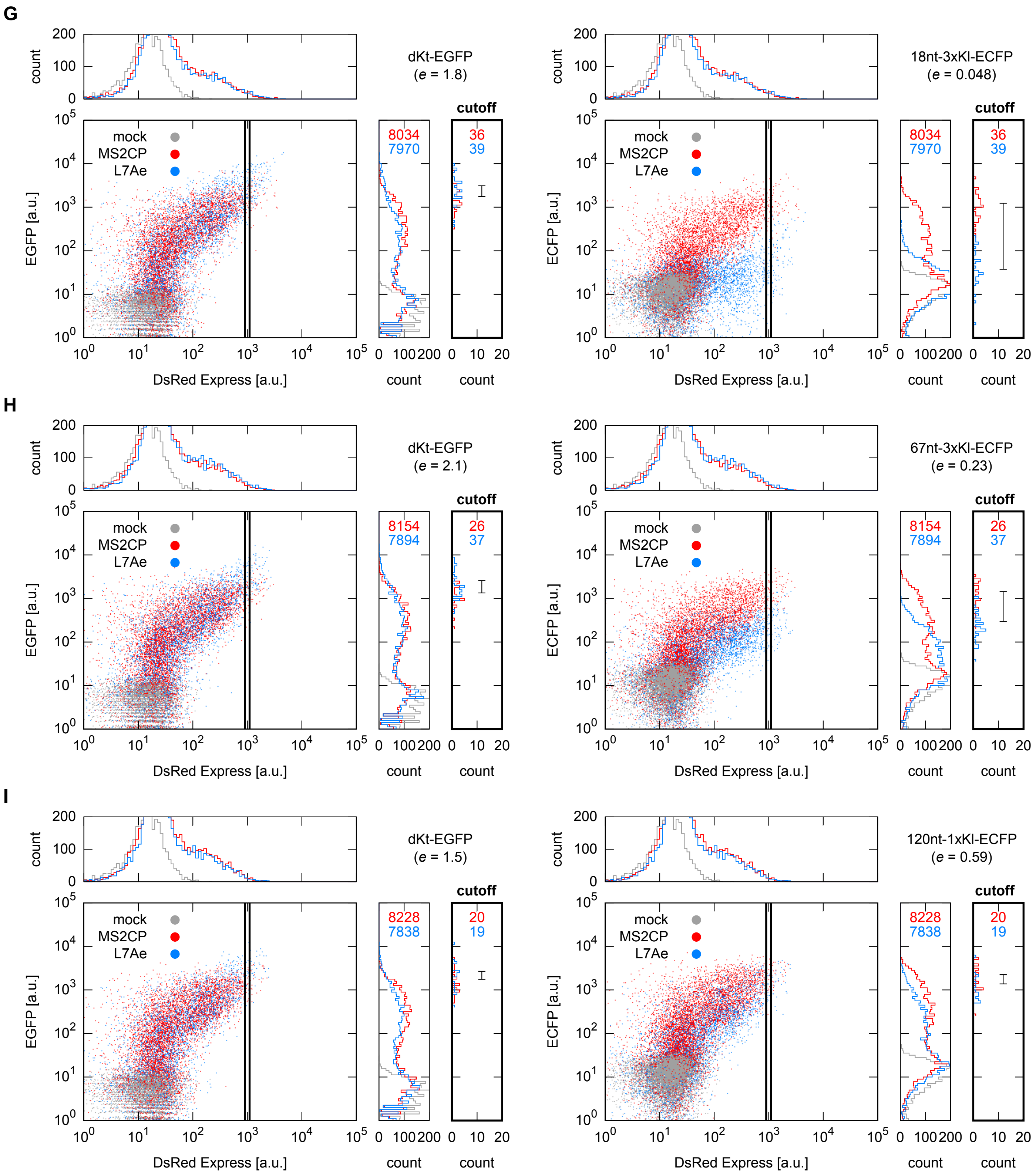
G
dKt-EGFP
(e = 1.8)
18nt-3xKl-ECFP
(e = 0.048)
mock
MS2CP
L7Ae
cutoff
8034
7970
36
39
H
dKt-EGFP
(e = 2.1)
67nt-3xKl-ECFP
(e = 0.23)
mock
MS2CP
L7Ae
cutoff
8154
7894
26
37
I
dKt-EGFP
(e = 1.5)
120nt-1xKl-ECFP
(e = 0.59)
mock
MS2CP
L7Ae
cutoff
8228
7838
20
19
count
EGFP [a.u.]
ECFP [a.u.]
DsRed Express [a.u.]

Supplementary Figure S3. Plots of a flow cytometric analysis presented in Figure 7. EGFP (left) or ECFP (right) outputs from co-transfected two constructs; Kt-EGFP and 18nt-3xKl-ECFP (**A**), Kt-EGFP and 67nt-3xKl-ECFP (**B**), Kt-EGFP and 120nt-1xKl-ECFP (**C**), Sp-Kt-EGFP and 18nt-3xKl-ECFP (**D**), Sp-Kt-EGFP and 67nt-3xKl-ECFP (**E**), Sp-Kt-EGFP and 120nt-1xKl-ECFP (**F**), dKt-EGFP and 18nt-3xKl-ECFP (**G**), dKt-EGFP and 67nt-3xKl-ECFP (**H**), and dKt-EGFP and 120nt-1xKl-ECFP (**I**), were shown as a function of DsRed-Express in the same manner as Figure 2. Representative results out of three independent experiments are shown, except translational efficiencies that were the average of the three experiments.

Supplementary Tables

Supplementary Table S1. Sequences of the 5' UTRs containing Kt.

Constructs [a]	Sequences of the 5' UTR [b]	Translational efficiency [c]
Kt-EGFP	UCAGAUCCGCUAGCGCUACCGGACUC*AGAUCU*GGGGCGUGAUCCGAAAGGUGACCC*GGAUCC*ACCGGUCGCCACC**AUG**	0.017 ± 0.0015
32nt-Kt		0.019 ± 0.0019
Sp-Kt-EGFP	UCAGAUCCGCUAGCGAUACACCGCAUCCGGCGCGGAUUGGCCUGAACUGCCAGCUGGCGCAGGUAGCAGAGCGGGUAAACUGGCUCGGAUUAGGGCCGCAAGAAAACUAUCCCGACCGCCUUACUGCCGCCUGUUUUGACCGCUGGGAUCUGCCAUUG*AGAUCU*GGGGCGUGAUCCGAAAGGUGACCC*GGAUCC*ACCGGUCGCCACC**AUG**	0.20 ± 0.051
164nt-Kt		0.23 ± 0.031
Kt-Sp-EGFP	UCAGAUCCGCUAGCGCUACCGGACUC*AGAUCU*GGGGCGUGAUCCGAAAGGUGACCC*GGAUCC*GAUCCCGUCGUUUUACAACGUCGUGACUGGGAAAACCCUGGCGUUACCCAACUUAAUCGCCUUGCAGCACAUCCCCCUUUCGCCAGCUGGCGUAAUAGCGAAGAGGCCCGCACCGAUCGCCCUUCCCAACAGUUGCGCAGCCUGACCGGUCGCCACC**AUG**	0.020 ± 0.00081
18nt-Kt [d]	UCAGAUCCGCUA*GGAUCU*GGGGCGUGAUCCGAAAGGUGACCC*GGAUCC*ACCGGUCGCCACC**AUG**	0.017 ± 0.0047
51nt-Kt	UCAGAUCCGCUAGCCGCCUGUUUUGACCGCUGGGAUCUGCCAUUG*AGAUCU*GGGGCGUGAUCCGAAAGGUGACCC*GGAUCC*ACCGGUCGCCACC**AUG**	0.026 ± 0.0065
67nt-Kt	UCAGAUCCGCUAGCCCGACCGCCUUACUGCCGCCUGUUUUGACCGCUGGGAUCUGCCAUUG*AGAUCU*GGGGCGUGAUCCGAAAGGUGACCC*GGAUCC*ACCGGUCGCCACC**AUG**	0.036 ± 0.0024
94nt-Kt	UCAGAUCCGCUAGCUCGGAUUAGGGCCGCAAGAAAACUAUCCCGACCGCCUUACUGCCGCCUGUUUUGACCGCUGGGAUCUGCCAUUG*AGAUCU*GGGGCGUGAUCCGAAAGGUGACCC*GGAUCC*ACCGGUCGCCACC**AUG**	0.094 ± 0.012
120nt-Kt	UCAGAUCCGCUAGCGCAGGUAGCAGAGCGGGUAAACUGGCUCGGAUUAGGGCCGCAAGAAAACUAUCCCGACCGCCUUACUGCCGCCUGUUUUGACCGCUGGGAUCUGCCAUUG*AGAUCU*GGGGCGUGAUCCGAAAGGUGACCC*GGAUCC*ACCGGUCGCCACC**AUG**	0.075 ± 0.019
145nt-Kt	UCAGAUCCGCUAGCGGAUUGGCCUGAACUGCCAGCUGGCGCAGGUAGCAGAGCGGGUAAACUGGCUCGGAUUAGGGCCGCAAGAAAACUAUCCCGACCGCCUUACUGCCGCCUGUUUUGACCGCUGGGAUCUGCCAUUG*AGAUCU*GGGGCGUGAUCCGAAAGGUGACCC*GGAUCC*ACCGGUCGCCACC**AUG**	0.18 ± 0.032
320nt-Kt	UCAGAUCCGCUAGCGAUACACCGCAUCCGGCGCGGAUUGGCCUGAACUGCCAGCUGGCGCAGGUAGCAGAGCGGGUAAACUGGCUCGGAUUAGGGCCGCAAGAAAACUAUCCCGACCGCCUUACUGCCGCCUGUUUUGACCGCUGGGAUCUGCCAUUGAGAUCCGAUCCCGUCGUUUUACAACGUCGUGACUGGGAAAACCCUGGCGUUACCCAACUUAAUCGCCUUGCAGCACAUCCCCCUUUCGCCAGCUGGCGUAAUAGCGAAGAGGCCCGCACCGAUCGCCCUUCCCAACAGUUGCGCAGCCUGACCGGU*AGAUCU*GGGGCGUGAUCCGAAAGGUGACCC*GGAUCC*ACCGGUCGCCACC**AUG**	0.24 ± 0.040

a ECFP was used as an output protein to determine the translational efficiency, except Kt-EGFP, Sp-Kt-EGFP, Kt-Sp-EGFP.
b Start codons are shown in bold. BglII and BamHI sites at the ends of Kt are shown in italics. Underlined nucleotides were altered to C in dKt construct.
c The average and the standard deviation of the three independent experiments are shown.
d The construction procedure resulted in a nucleotide change in the BglII site (AGAUCU) located at the 5' end of Kt to GGAUCU.

Supplementary Table S2. Sequences of the 5' UTRs containing Kl, Kl2, MS2SL, or Fr15.

construct	Sequences of the 5' UTR [a]	Translational efficiency
Kl2	UCAGAUCCGCUAGCGCUACCGGACUC*AGAUCC*GGACGUACGUGUGAACGGUGAUCACGUACGCCG*AGAUCC*ACCGGUCGCCACC**AUG**	0.84 ± 0.17
2xKl2	UCAGAUCCGCUAGCGCUACCGGACUC*AGAUCC*GGACGUACGUGUGAACGGUGAUCACGUACGCCGAGAUCCGGACGUACGUGUGAACGGUGAUCACGUACGCCG*AGAUCC*ACCGGUCGCCACC**AUG**	0.093 ± 0.0064
18nt-1xKl	UCAGAUCCGCUA*GGAUCC*GGGUGUGAACGGUGAUCACCCG*AGAUCC*ACCGGUCGCCACC**AUG**	0.28 ± 0.071
18nt-2xKl	UCAGAUCCGCUA*GGAUCC*GGGUGUGAACGGUGAUCACCCGAGAUCCGGGUGUGAACGGUGAUCACCCG*AGAUCC*ACCGGUCGCCACC**AUG**	0.084 ± 0.014
18nt-3xKl	UCAGAUCCGCUA*GGAUCC*GGGUGUGAACGGUGAUCACCCGAGAUCCGGGUGUGAACGGUGAUCACCCGAGAUCCGGGUGUGAACGGUGAUCACCCG*AGAUCC*ACCGGUCGCCACC**AUG**	0.039 ± 0.013
18nt-4xKl	UCAGAUCCGCUA*GGAUCC*GGGUGUGAACGGUGAUCACCCGAGAUCCGGGUGUGAACGGUGAUCACCCGAGAUCCGGGUGUGAACGGUGAUCACCCGAGAUCCGGGUGUGAACGGUGAUCACCCG*AGAUCC*ACCGGUCGCCACC**AUG**	0.069 ± 0.011
67nt-1xKl	UCAGAUCCGCUAGCCCGACCGCCUUACUGCCGCCUGUUUGACCGCUGGGAUCUGCCAUUG*AGAUCC*GGGUGUGAACGGUGAUCACCCG*AGAUCC*ACCGGUCGCCACC**AUG**	0.41 ± 0.060
67nt-2xKl	UCAGAUCCGCUAGCCCGACCGCCUUACUGCCGCCUGUUUGACCGCUGGGAUCUGCCAUUG*AGAUCC*GGGUGUGAACGGUGAUCACCCGAGAUCCGGGUGUGAACGGUGAUCACCCG*AGAUCC*ACCGGUCGCCACC**AUG**	0.25 ± 0.027
67nt-3xKl	UCAGAUCCGCUAGCCCGACCGCCUUACUGCCGCCUGUUUGACCGCUGGGAUCUGCCAUUG*AGAUCC*GGGUGUGAACGGUGAUCACCCGAGAUCCGGGUGUGAACGGUGAUCACCCGAGAUCCGGGUGUGAACGGUGAUCACCCG*AGAUCC*ACCGGUCGCCACC**AUG**	0.15 ± 0.015
67nt-4xKl	UCAGAUCCGCUAGCCCGACCGCCUUACUGCCGCCUGUUUGACCGCUGGGAUCUGCCAUUG*AGAUCC*GGGUGUGAACGGUGAUCACCCGAGAUCCGGGUGUGAACGGUGAUCACCCGAGAUCCGGGUGUGAACGGUGAUCACCCGAGAUCCGGGUGUGAACGGUGAUCACCCG*AGAUCC*ACCGGUCGCCACC**AUG**	0.12 ± 0.018

120nt-1xKl	UCAGAUCCGCUAGCGCAGGUAGCAGAGCGGGUAAACUGGCUCGGAUUAGGGCCGCAAGAAAACUAUCCCGACCGCCUUACUGCCGCCUGUUUUGACCGCUGGGAUCUGCCAUUG*AGAUCC*GGGUGUGAACGGUGAUCACCCG*AGAUCC*ACCGGUCGCCACC**AUG**	0.67 ± 0.059
120nt-2xKl	UCAGAUCCGCUAGCGCAGGUAGCAGAGCGGGUAAACUGGCUCGGAUUAGGGCCGCAAGAAAACUAUCCCGACCGCCUUACUGCCGCCUGUUUUGACCGCUGGGAUCUGCCAUUG*AGAUCC*GGGUGUGAACGGUGAUCACCCGAGAUCCGGGUGUGAACGGUGAUCACCCG*AGAUCC*ACCGGUCGCCACC**AUG**	0.31 ± 0.031
120nt-3xKl	UCAGAUCCGCUAGCGCAGGUAGCAGAGCGGGUAAACUGGCUCGGAUUAGGGCCGCAAGAAAACUAUCCCGACCGCCUUACUGCCGCCUGUUUUGACCGCUGGGAUCUGCCAUUG*AGAUCC*GGGUGUGAACGGUGAUCACCCGAGAUCCGGGUGUGAACGGUGAUCACCCGAGAUCCGGGUGUGAACGGUGAUCACCCG*AGAUCC*ACCGGUCGCCACC**AUG**	0.19 ± 0.015
120nt-4xKl	UCAGAUCCGCUAGCGCAGGUAGCAGAGCGGGUAAACUGGCUCGGAUUAGGGCCGCAAGAAAACUAUCCCGACCGCCUUACUGCCGCCUGUUUUGACCGCUGGGAUCUGCCAUUG*AGAUCC*GGGUGUGAACGGUGAUCACCCGAGAUCCGGGUGUGAACGGUGAUCACCCGAGAUCCGGGUGUGAACGGUGAUCACCCGAGAUCCGGGUGUGAACGGUGAUCACCCG*AGAUCC*ACCGGUCGCCACC**AUG**	0.12 ± 0.0052
164nt-1xKl	UCAGAUCCGCUAGCGAUACACCGCAUCCGGCGCGGAUUGGCCUGAACUGCCAGCUGGCGCAGGUAGCAGAGCGGGUAAACUGGCUCGGAUUAGGGCCGCAAGAAAACUAUCCCGACCGCCUUACUGCCGCCUGUUUUGACCGCUGGGAUCUGCCAUUG*AGAUCC*GGGUGUGAACGGUGAUCACCCG*AGAUCC*ACCGGUCGCCACC**AUG**	0.58 ± 0.087
164nt-2xKl	UCAGAUCCGCUAGCGAUACACCGCAUCCGGCGCGGAUUGGCCUGAACUGCCAGCUGGCGCAGGUAGCAGAGCGGGUAAACUGGCUCGGAUUAGGGCCGCAAGAAAACUAUCCCGACCGCCUUACUGCCGCCUGUUUUGACCGCUGGGAUCUGCCAUUG*AGAUCC*GGGUGUGAACGGUGAUCACCCGAGAUCCGGGUGUGAACGGUGAUCACCCG*AGAUCC*ACCGGUCGCCACC**AUG**	0.34 ± 0.044
164nt-3xKl	UCAGAUCCGCUAGCGAUACACCGCAUCCGGCGCGGAUUGGCCUGAACUGCCAGCUGGCGCAGGUAGCAGAGCGGGUAAACUGGCUCGGAUUAGGGCCGCAAGAAAACUAUCCCGACCGCCUUACUGCCGCCUGUUUUGACCGCUGGGAUCUGCCAUUG*AGAUCC*GGGUGUGAACGGUGAUCACCCGAGAUCCGGGUGUGAACGGUGAUCACCCGAGAUCCGGGUGUGAACGGUGAUCACCCG*AGAUCC*ACCGGUCGCCACC**AUG**	0.21 ± 0.021
164nt-4xKl	UCAGAUCCGCUAGCGAUACACCGCAUCCGGCGCGGAUUGGCCUGAACUGCCAGCUGGCGCAGGUAGCAGAGCGGGUAAACUGGCUCGGAUUAGGGCCGCAAGAAAACUAUCCCGACCGCCUUACUGCCGCCUGUUUUGACCGCUGGGAUCUGCCAUUG*AGAUCC*GGGUGUGAACGGUGAUCACCCGAGAUCCGGGUGUGAACGGUGAUCACCCGAGAUCCGGGUGUGAACGGUGAUCACCCGAGAUCCGGGUGUGAACGGUGAUCACCCG*AGAUCC*ACCGGUCGCCACC**AUG**	0.17 ± 0.012
18nt -1xMS2SL	UCAGAUCCGCUA*GGAUCC*GGUGAGGAUCACCCAUCG*AGAUCC*ACCGGUCGCCACC**AUG**	0.48 ± 0.16
18nt -2xMS2SL	UCAGAUCCGCUA*GGAUCC*GGUGAGGAUCACCCAUCGAGAUCCGGUGAGGAUCACCCAUCG*AGAUCC*ACCGGUCGCCACC**AUG**	0.18 ± 0.042

67nt -1xMS2SL	UCAGAUCCGCUAGCCCGACCGCCUUACUGCCGCCUGUUUUGACCGCUGGGAUCUGCCAUUG*AGAUCC*GGUGAGGAUCACCCAUCG*AGAUCC*ACCGGUCGCCACC**AUG**	1.0 ± 0.35
67nt -2xMS2SL	UCAGAUCCGCUAGCCCGACCGCCUUACUGCCGCCUGUUUUGACCGCUGGGAUCUGCCAUUG*AGAUCC*GGUGAGGAUCACCCAUCGAGAUCCGGUGAGGAUCACCCAUCG*AGAUCC*ACCGGUCGCCACC**AUG**	0.67 ± 0.22
18nt-1xFr15	UCAGAUCCGCUA*GGAUCC*UCGGUCGAAAGACUUGAGGGCAGGAGAGGACUUCGGUCUGGCCUGCACCUGACG*AGAUCC*ACCGGUCGCCACC**AUG**	0.31 ± 0.032
18nt-2xFr15	UCAGAUCCGCUA*GGAUCC*UCGGUCGAAAGACUUGAGGGCAGGAGAGGACUUCGGUCUGGCCUGCACCUGACGAGAUCCUCGGUCGAAAGACUUGAGGGCAGGAGAGGACUUCGGUCUGGCCUGCACCUGACG*AGAUCC*ACCGGUCGCCACC**AUG**	0.16 ± 0.0082
67nt-1xFr15	UCAGAUCCGCUAGCCCGACCGCCUUACUGCCGCCUGUUUUGACCGCUGGGAUCUGCCAUUG*AGAUCC*UCGGUCGAAAGACUUGAGGGCAGGAGAGGACUUCGGUCUGGCCUGCACCUGACG*AGAUCC*ACCGGUCGCCACC**AUG**	0.45 ± 0.10
67nt-2xFr15	UCAGAUCCGCUAGCCCGACCGCCUUACUGCCGCCUGUUUUGACCGCUGGGAUCUGCCAUUG*AGAUCC*UCGGUCGAAAGACUUGAGGGCAGGAGAGGACUUCGGUCUGGCCUGCACCUGACGAGAUCCUCGGUCGAAAGACUUGAGGGCAGGAGAGGACUUCGGUCUGGCCUGCACCUGACG*AGAUCC*ACCGGUCGCCACC**AUG**	0.22 ± 0.030

a Sequences are shown as in Supplementary Table S1 except that indicated RNA motifs are presented with underlined letters.

Supplementary Table S3. Verified combinations of an RNA motif for translation repression and an output protein.

RNA motif	Output protein	Reference
K-turn	EGFP, DsRed	Saito et al, *Nat. Chem. Biol.*, 2012 (18)
	Bcl-xL, Bim, FADD	Saito et al, *Nat. Commun.*, 2011 (19)
	d2EYFP, DsRed	Ausänder et al, *Nature*, 2012 (15)
K-loop	EGFP, L7Ae-ECFP	Stapleton et al, *ACS Synth. Biol.*, 2012 (20)
MS2 stem loop	Luciferase	Stripecke et al, *Mol. Cell Biol.*, 1994 (22)
	YFP	Nie et al, *Nucleic Acids Res.*, 2006 (23)
	d2EYFP, DsRed	Ausänder et al, *Nature*, 2012 (15)
Fr15	ECFP	this study
U1A	Luciferase	Stripecke et al, *Mol. Cell Biol.*, 1994 (20)
TBS, IRE, vault RNA	YFP	Nie et al, *Nucleic Acids Res.*, 2006 (22)
